# 8‐Prenylnaringenin tissue distribution and pharmacokinetics in mice and its binding to human serum albumin and cellular uptake in human embryonic kidney cells

**DOI:** 10.1002/fsn3.2733

**Published:** 2022-01-22

**Authors:** Yoshiaki Tanaka, Hitomi Okuyama, Miyu Nishikawa, Shin‐ichi Ikushiro, Mayumi Ikeda, Yu Ishima, Yuichi Ukawa, Kenichi Oe, Junji Terao, Rie Mukai

**Affiliations:** ^1^ Department of Food Science Graduate School of Biomedical Sciences Tokushima University Tokushima Japan; ^2^ Department of Food Science Graduate School of Technology, Industrial and Social Sciences Tokushima University Tokushima Japan; ^3^ Department of Biotechnology Faculty of Engineering Toyama Prefectural University Toyama Japan; ^4^ Department of Pharmacokinetics and Biopharmaceutics Institute of Biomedical Sciences Tokushima University Tokushima Japan; ^5^ Healthcare SBU Business Strategy Business Planning Daicel Corporation Tokyo Japan; ^6^ Healthcare SBU Business Strategy, R&D Daicel Corporation Niigata Japan; ^7^ Faculty of Clinical Nutrition and Dietetics Konan Women's University Hyogo Japan

**Keywords:** 8‐prenylnaringenin, naringenin, pharmacokinetics, serum albumin, tissue accumulation

## Abstract

8‐Prenylnaringenin (8‐PN), a hop flavonoid, is a promising food substance with health benefits. Compared with nonprenylated naringenin, 8‐PN exhibits stronger estrogenic activity and prevents muscle atrophy. Moreover, 8‐PN prevents hot flushes and bone loss. Considering that prenylation reportedly improves the bioavailability of flavonoids, we compared the parameters related to the bioavailability [pharmacokinetics and tissue distribution in C57/BL6 mice, binding affinity to human serum albumin (HSA), and cellular uptake in HEK293 cells] of 8‐PN and its mother (non‐prenylated) compound naringenin. C57/BL6 mice were fed an 8‐PN or naringenin mixed diet for 22 days. The amount of 8‐PN (nmol/g tissue) in the kidneys (16.8 ± 9.20), liver (14.8 ± 2.58), muscles (3.33 ± 0.60), lungs (2.07 ± 0.68), pancreas (1.80 ± 0.38), heart (1.71 ± 0.27), spleen (1.36 ± 0.29), and brain (0.31 ± 0.09) was higher than that of naringenin. A pharmacokinetic study in mice demonstrated that the *C*_max_ of 8‐PN (50 mg/kg body weight) was lower than that of naringenin; however, the plasma concentration of 8‐PN 8 h after ingestion was higher than that of naringenin. The binding affinity of 8‐PN to HSA and cellular uptake in HEK293 cells were higher than those of naringenin. 8‐PN bioavailability features assessed in mouse or human model experiments were obviously different from those of naringenin.

## Graphical abstract

This study aimed to compare the parameters related to the bioavailability of 8‐PN and its mother (nonprenylated) compound naringenin. The tissue distribution of 8‐PN in mice was higher than that of naringenin. Compared with naringenin, 8‐PN showed a stronger affinity to human serum albumin and higher cellular uptake in human embryonic kidney cells.

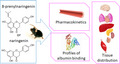

## 1 INTRODUCTION

*Humulus lupulus* L. (hop) contains prenylflavonoids, including 8‐prenylnaringenin (8‐PN), 6‐prenylnaringenin, isoxanthohumol (IX), and xanthohumol (XN) (Stevens et al., 1999). These flavonoids are believed to be the active ingredients of hops, which is used in hormone replacement therapy for menopausal women (Erkkola et al., 2010). Specifically, 8‐PN has been found to exhibit the strongest estrogenic activity among the hop flavonoids contributing to its therapeutic effect (Milligan et al., 2000). It has also been demonstrated to suppress bone loss and uterine atrophy (Hümpel et al., 2005) and contribute to the maintenance of uterine weight, endothelia, and 17β‐estradiol in ovariectomized female rats (Diel et al., 2004). In addition to estrogenic activity, 8‐PN also reportedly promotes protein synthesis in the skeletal muscles of male and female mice (Mukai et al., 2012, 2016) and prevents diabetes in male mice by regulating vascular endothelial growth factor signaling in the kidneys and left ventricle (Costa et al., 2017) or by suppressing oxidative stress in the liver and kidneys (Luís et al., 2019). These reports imply that 8‐PN can improve health conditions, regardless of sex, by affecting the physiology of target organs.

Diet, beverages, and supplements containing hop extract are the major sources of 8‐PN and its precursor IX (Stevens et al., 1999). IX is converted to 8‐PN via demethylation by either human liver CYP1A2 (Guo et al., 2006) or gut microflora in the human intestine (Bolca et al., 2007; Possemiers et al., 2006). As 8‐PN undergoes phase II metabolism during epithelial absorption in the small intestine, both glucuronide and sulfate conjugates of 8‐PN have been detected in Caco‐2 cells (Nikolic et al., 2006). Twelve phase I metabolites of 8‐PN have also been detected in human microsomes (Nikolic et al., 2004).

The bioavailability of 8‐PN has become a key research topic for evaluating its influence on health. Rad et al. (2006) demonstrated that 8‐PN was rapidly absorbed into the bloodstream of postmenopausal women 1–1.5 h after oral administration, mostly as conjugated metabolites with small amounts of aglycone. The pharmacokinetic properties of 8‐PN reveal that this compound also undergoes enterohepatic recirculation, prolonging its mean residence time (Rad et al., 2006). Additionally, the conjugated metabolites and aglycone derived from 8‐PN are primarily excreted in the feces and bile (Rad et al., 2006). In fact, tissue accumulation of 8‐PN, XN, and IX has been reported in the adipose and glandular tissues of breast tissue following hop supplementation in women (Bolca et al., 2010). Furthermore, XN and 8‐PN were detected in the liver and mammary tissues of rats after daily injections of XN for 4 days (Dietz et al., 2013). However, the distribution of 8‐PN in other organs and tissues has not been fully investigated.

Investigating the interaction between phytochemicals and protein is one of the interesting avenues that helps to understand their pharmacokinetics and/or pharmacodynamics. The plasma proteins [e.g., human serum albumin (HSA)] and DNA are reported to interact with drugs, hormones, and phytochemicals (Danesh et al., 2018; Dareini et al., 2020; Mokaberi et al., 2021; Sharifi‐Rad et al., 2021; Zare‐Feizabadi et al., 2021). It has been also reported that several flavonoids, including naringenin, bind to has (Bolli et al., 2010; Cao et al., 2019; Tu et al., 2015; Zinellu et al., 2015). It is widely accepted in pharmaceutical research that the tissue distribution, metabolism, and efficacy of phytochemicals or drugs can be altered based on their affinity with HSA. Although much information on the biological activity of 8‐PN exists, its interaction with HSA has not yet been investigated.

**FIGURE 1:**
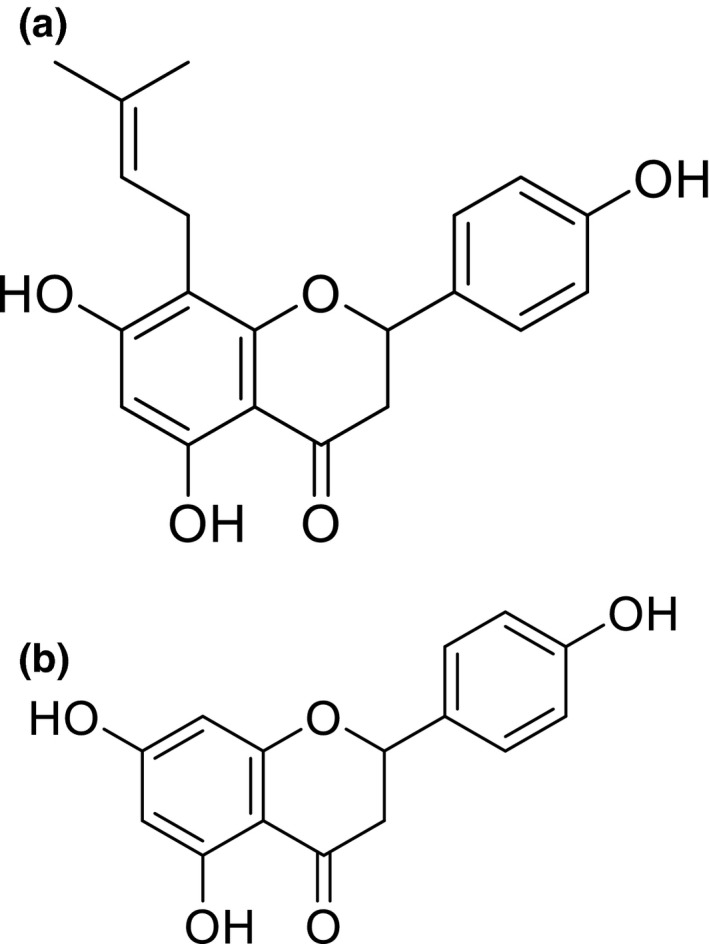
Structures of 8‐PN (a) and naringenin (b)

Previously, we reported that prenylation is a structural modification that affects the pharmacokinetics and tissue accumulation of flavonoids using several experimental models [mice (Mukai et al., 2012, 2013), rats (Mukai et al., 2013) and human (Mukai et al., 2013) and mouse cell lines (Mukai et al., 2016)]. The chemical structures of 8‐PN and naringenin are shown in Figure 1. Higher levels of 8‐PN were detected in the gastrocnemius muscles of mice than those of naringenin after 22 days of hop supplementation (Mukai et al., 2012). Furthermore, the *C*_max_ of 8‐PN in plasma was much lower than that of naringenin (Mukai et al., 2012). It was speculated that the transport of 8‐PN from blood to tissue or from tissue to blood was affected by naringenin prenylation. We also reported that prenylation of quercetin enhances its cellular uptake and reduces its excretion from both Caco‐2 and C2C12 cells (Mukai, 2018; Mukai et al., 2013), suggesting that other prenylflavonoids may be absorbed to a greater degree in tissues and organs than that seen with nonprenylated flavonoids. This study aimed to elucidate the pharmacokinetics and tissue distribution of 8‐PN in comparison with those of naringenin. Hence, this study investigates the tissues or organs in which the health‐promoting effect of 8‐PN is seen. In addition, comparison with 8‐PN and naringenin may explain the importance of prenylation to flavonoid aglycone on tissue distribution, and when combined with the knowledge of the effect of prenylation on pharmacokinetics of flavonoid, our findings can contribute to the development of prenylflavonoids as a nutritional supplement that promotes health.

## 2 EXPERIMENTAL PROCEDURE

### 2.1 Materials

Naringenin, eriodictyol, and phenylbutazone were obtained from Tokyo Chemical Industry Co., Ltd. (TCI). Ibuprofen was purchased from FUJIFILM Wako Pure Chemical Corp. Pentamethyl quercetin and kaempferol were obtained from Extrasynthase. Additionally, 8‐PN and 8‐prenylhesperetin were synthesized by our research team (Kawamura et al., 2012). Naringenin 4′‐glucuronide (N4′G) was obtained from Cayman Chemical Company. The glucuronides, namely, naringenin 7‐glucuronide (N7G), 8‐PN 7‐glucuronide (8‐PN‐7G), and 8‐PN 4′‐glucuronide (8‐PN‐4′G) were enzymatically prepared by incubating the parent compounds with resting yeast cells co‐expressing uridine diphosphate‐glucose dehydrogenase and suitable mammalian UDP‐glucuronosyltransferase isoforms (mouse Ugt2b1 for N7G, monkey UGT1A1 for 8‐PN‐7G, and rat UGT2B1 for 8‐PN‐4′G) as previously described with slight modifications (Ikushiro et al., 2016; Nakamura et al., 2018; Tanaka et al., 2018).

### 2.2 Dosage information

We planned the nutritional composition and feeding period according to previous similar research (Andres‐Lacueva et al., 2012; Bieger et al., 2008; Boer et al., 2005; Mukai et al., 2013; Takumi et al., 2011). Briefly, in the evaluation of tissue distribution (Section 2.3), we prepared 8‐PN or naringenin mixed diet. 8‐PN and naringenin were contained in the diet at 0.2% (w/w) and 0.17% (w/w), respectively. Since the molecular weight (8‐PN: 340.4; naringenin: 272.3) and the purity (8‐PN: 95%; naringenin: 90%) of these nutrients were different, we applied different concentrations (w/w) for each diet. Finally, the same concentration of both nutrients was adjusted to 5.6 mmol compound/kg diet. Though this dose concentration may not be achieved through regular diet or supplementation, this concentration was used in accordance with previous studies that evaluated the tissue distribution of flavonoids (0.2% [w/w]) (Mukai et al., 2013; Takumi et al., 2011). The mice were fed each diet via normal oral feeding for 22 days. In the pharmacokinetic study (Section 2.5), we administered each nutrient to mice at a dose of 50 mg/kg body weight (BW) via oral gavage after 18 h of starvation. Although this dose concentration may not reflect regular diet or supplementation, this or higher concentrations have been applied for pharmacokinetic study of flavonoids (Bai et al., 2020; Hung et al., 2018; Mukai et al., 2013).

### 2.3 Evaluation of tissue distribution

All experimental protocols were approved by the Committee on Animal Experiments of Tokushima University (approval number: 11013). All efforts were made to minimize animal suffering. Seven‐week‐old male C57/BL6 mice (Japan SLC) were housed in a room maintained at 23 ± 1°C under a 12‐h/12‐h light/dark cycle. Diets consisted of 8‐PN or naringenin (0.56 mmol/kg diet) mixed with AIN‐93M (AIN‐93M; Oriental Yeast Company), with the cellulose contents reduced to adjust the composition of other nutrients. The mice (*n* = 7 or 8) were fed these diets via normal oral feeding for 22 days and allowed free access to water. The liver, kidneys, quadricep muscles, pancreas, lungs, brain, spleen, and heart were collected from the mice under anesthesia and weighed. Before freezing, the liver tissues were flushed with ice‐cold phosphate‐buffered saline (PBS, pH 7.4) to avoid blood contamination. All samples were stored at −80°C under N_2_ gas until extraction of flavonoid.

### 2.4 Sample preparation for the determination of tissue accumulation

Sample preparation was performed according to a previous report (Mukai et al., 2013). Each tissue was homogenized in PBS on ice (nine times the volume of the tissue wet weight) using a Teflon homogenizer (As One). To determine total 8‐PN and naringenin (conjugated metabolites plus aglycone) amount in the tissues, homogenates were incubated with 50 mM ascorbic acid (0.2 times volume of PBS) and 100 U/100 µl β‐glucuronidase type H‐1 (Sigma‐Aldrich), which possessed β‐glucuronidase and sulfatase activity, in acetic acid‐sodium acetate buffer (pH 5.0, same volume as PBS) for 2 h at 37°C. Before extraction, 100 pmol of 8‐prenylhesperetin or eriodictyol was added to the hydrolysates as the internal standard for 8‐PN or naringenin, respectively. The hydrolysates were then subjected to extraction three times with equal volumes of ethyl acetate and evaporated using a centrifugal evaporator (CVE‐100; Tokyo Rikakikai). The extracts were dissolved in 50 µl of methanol containing 0.5% phosphoric acid. Then, 20 µl from each sample was injected into the HPLC–UV system.

### 2.5 Pharmacokinetics of 8‐PN and naringenin

Seven‐week‐old male C57/BL6 mice (Japan SLC) were housed in a room maintained at 23 ± 1°C under a 12‐h/12‐h light/dark cycle with free access to a commercial diet (AIN‐93M) and water for 1 week. They were deprived of food 18 h before administration, but had free access to water. Solutions of 8‐PN or naringenin dissolved in propylene glycol were administered (50 mg/kg BW) to the mice via oral gavage. Blood samples (approximately 50 μl) were then collected from tail veins 0.5, 1, 2, 4, 8, 24, and 48 h after administration. Plasma was isolated by centrifugation at 9000 *g* for 10 min at 4°C and stored at −80°C under N_2_ gas until use.

### 2.6 Sample preparation for the determination of plasma concentration

The concentrations of the conjugated metabolites and aglycones of each flavonoid were quantified as described previously (Mukai et al., 2012). Briefly, the plasma (10 µl) was incubated with 100 U of β‐glucuronidase type H‐1 (Sigma‐Aldrich) prepared in 0.1 M sodium acetate buffer (pH 5.0; 90 µl) and 50 mM ascorbic acid (20 µl) for 45 min. Next, 100 pmol of pentamethyl quercetin or kaempferol was added to the samples as the internal standard for 8‐PN or naringenin, respectively. Liberated aglycone was extracted using ethyl acetate and evaporated with a centrifugal evaporator. The pellets were dissolved in 75 µl methanol containing 0.5% phosphoric acid. A total of 25 µl of each sample was injected into the HPLC–UV detection system.

### 2.7 Binding properties of 8‐PN and its conjugated metabolites with site‐selective HSA‐binding fluorescent probes

The binding properties of flavonoids to HSA were assessed using a fluorescence competitive binding assay with the fluorescent dyes dansylamide (DNSA; specifically binds to site I of HSA; TCI) and BD140 (specifically binds to site II of HSA; TCI) for multiplex drug‐site mapping on HSA (Er et al., 2013). A fluorescent dye cocktail containing 0.33 mM BD140 and 1 mM DNSA was prepared in DMSO. Defatted HSA (A3782‐1G; Sigma‐Aldrich) was dissolved in phosphate buffer (pH 7.4) at a concentration of 0.67 mg/ml. Each flavonoid was dissolved in DMSO (stock solution) at 100 times the test concentration. Subsequently, 637 µl HSA solution, 6.5 µl fluorescent dye cocktail, and 6.5 µl flavonoid were mixed and divided into three wells on a 96‐well multi‐black plate. The final concentration of flavonoids was 1–100 μM. The fluorescence intensities of DNSA (Ex: 360 nm, Em: 465 nm) and BD140 (Ex: 535 nm, Em: 590 nm) were measured using a TECAN infinite M200 (Tecan Group Ltd.). The HSA‐binding affinities of the flavonoids were evaluated by measuring the competitive inhibition rate of the probes binding to flavonoids according to Equation (1):

(1) $$\text{Flavonoid}\;\text{inhibition}\;\text{percentage}\;\left(\%\right)=\frac{F_{1}‐F_{2}}{F_{1}}\times 100,$$

where *F*_1_ is the fluorescence intensity of DMSO and *F*_2_ represents the fluorescence intensity of each flavonoid.

The inner filter effect is involved in fluorescent spectroscopy (Askari et al., 2021). This effect was estimated as described here. Since the fluorescent probes exhibit fluorescence when they interact with HSA, we cannot evaluate the quenching effect of flavonoids on these probes. On the other hand, the *λ*_max_ of naringenin or 8‐PN [naringenin: 213, 225, and 289 nm and 8‐PN: 292 nm (referred by Cayman Chemical)] were different from the excitation wavelength for DNSA and BD140.

### 2.8 Cell culture and sample preparation for cellular uptake

Human embryonic kidney 293 (HEK293) cells (CRL‐1573, ATCC, DC, NW.) were maintained in Dulbecco's modified Eagle's medium (D5796; Sigma‐Aldrich) supplemented with 10% fetal bovine serum, 100 U/ml penicillin, 100 μg/ml streptomycin, and 2 mM l‐glutamine at 37°C in a humidified atmosphere containing 5% CO_2_. Cells seeded on a 60‐mm dish (1.0 × 10^5^ cells/dish) were cultured until confluent. Cells were then incubated with 100 μM NaN_3_ for 15 min before flavonoid treatment. Subsequently, cells were treated with 10 μM 8‐PN or naringenin for 1 h. After incubation, cells were washed twice with ice‐cold Hanks’ balanced salt solution (HBSS, pH 7.3) and scraped from the dish. After centrifugation (21,500 *g* for 10 min at 4°C), the supernatant was aspirated and 330 μl of HBSS was added to the cell pellet. Cell homogenate was obtained using sonication. The homogenate was divided into two portions, the first was used to analyze protein concentration, whereas the second was used for flavonoid extraction. The protein concentration of cell homogenate was measured using the Bradford assay. Another sample underwent deconjugation with β‐glucuronidase type H‐1 according to a previous report (Mukai et al., 2013). Before extraction, 100 pmol of 8‐prenylhesperetin (for 8‐PN) or eriodictyol (for naringenin) was added to cell homogenate as the internal standard. The homogenates were then subjected to extraction thrice in ethyl acetate using sonication for 1 min on an Astrason XL2020 Ultrasonic Processor (Heat Systems‐Ultrasonic) at level 10. After centrifugation (9000 *g* for 10 min at 4°C), the supernatants were collected, evaporated, and dissolved in 50 µl methanol containing 0.5% phosphoric acid. A total of 20 µl of each sample was injected into the HPLC–UV system.

### 2.9 HPLC analysis

HPLC was performed as previously described (Mukai et al., 2012). The 8‐PN and naringenin amounts in the tissues, plasma, and cells were analyzed via HPLC–UV detection under a *λ*_max_ value of 292 nm (SPD‐10AV; Shimadzu) with a Cadenza CD‐C18 HPLC column (3 µm, 4.6 × 150 mm; Imtact). In the mobile phase, solvent A was 0.5% phosphoric acid and solvent B was methanol containing 0.5% phosphoric acid. The B values for 8‐PN and naringenin detection were set at 65% and 43%, respectively. The flow rate was set at 1.0 ml/min.

8‐PN and naringenin amounts were determined using an internal standard method. They were identified from their retention times against those of respective standard compounds. We had confirmed that there were no apparent peaks comparable to 8‐PN or naringenin in chromatograms from tissue from nonfed mice (data not shown). The peak limit of detection (LOD) of was defined using the chromatogram. Since a clear peak was actually detected for 8‐PN over 2 pmol (0.1 μM), and for naringenin over 4 pmol (0.2 μM) based on visual evaluation, we determined these concentrations were the LODs for each flavonoid. We applied two calibration curves at the concentration ratio range of 8‐PN/8‐PH at 0.05–15 (*R* ^2^ = .9997) for low levels of 8‐PN or 1.5–250 (*R* ^2^ = .9999) for high levels of 8‐PN. Calibration curve at the concentration ratio range of naringenin/eriodictyol at 0.1–50 showed linearity (*R* ^2^ = .9997). The tested recovery rate and inter‐day and intra‐day variability (CV) are listed in Table 1.

**TABLE 1:** HPLC validation

	Naringenin	8‐PN
Recovery^a^ (%)		
Kidney	69 ± 11	110 ± 17
Liver	95 ± 19	123 ± 29
Muscle	**—**	118 ± 17
Lung	**—**	80 ± 703
Pancreas	**—**	93 ± 7.8
Heart	**—**	112 ± 0.8
Spleen	**—**	101 ± 17
Brain	**—**	59 ± 54
Intra‐day CV^b^ %	2.5	3.6
Inter‐day CV^b^ %	6.9	2.4

Note

**—**, not analyzed.

^a^ Values are presented as the means ± SD (*n* = 3). 8‐PN or naringenin (100 pmol) was added to each tissue homogenate and extracted based on the sample preparation method.

^b^ These CV were determined by comparing different injections on the same day (*n* = 3, the intra‐day) or different injections on different days (*n* = 3, the inter‐day).

### 2.10 Statistical analyses

Data are shown as the mean ± standard error (SE). Data were analyzed using the Mann–Whitney *U* test (*p* < .05). All statistical analyses were performed using Excel Tokei Ver. 7.0 for Windows (ESUMI Co., Ltd.).

## 3 RESULTS

### 3.1 8‐PN and naringenin tissue accumulation

The average daily food intake of the 8‐PN and naringenin diet groups during the feeding period was 4.4 and 4.8 g/day/mouse, respectively. The daily consumption of 8‐PN and naringenin in the 8‐PN and naringenin diet groups was approximately 24.6 and 27.0 µmol/day/mouse, respectively. Moreover, the amount of 8‐PN was 10 times more than that of naringenin in the kidneys, muscles, heart, and brain tissues (Table 2). Compared to that of naringenin, the amount of 8‐PN was higher in the other tissues as well. For liver tissues, we calculated the ratio of aglycone to the subtotal of aglycone and deconjugated metabolites (Table 3), yielding an average of 11.7%.

**TABLE 2:** Tissue distribution of 8‐PN and naringenin

Tissue source	Naringenin	8‐PN
	nmol/g wet tissue	
Kidney	1.32 ± 0.65	16.8 ± 9.20**
Liver	2.18 ± 2.89	14.8 ± 2.58**
Muscle	0.14 ± 0.03	3.33 ± 0.60**
Lung	0.35 ± 0.17	2.07 ± 0.68**
Pancreas	0.19 ± 0.07	1.80 ± 0.38**
Heart	0.16 ± 0.07	1.71 ± 0.27**
Spleen	0.20 ± 0.06	1.36 ± 0.29**
Brain	0.08 ± 0.02	0.31 ± 0.09

Note

Values are represented as the mean ± SE. Livers, kidneys, muscles, pancreases, lungs, and hearts of naringenin‐fed mice (*n* = 8). Brains and spleens of naringenin‐fed mice (*n* = 7). Tissues, except for the heart, from 8‐PN fed mice (*n* = 7). The hearts of 8‐PN fed mice (*n* = 6). Asterisks indicate significant differences between naringenin and 8‐PN as analyzed by using the Mann–Whitney *U* test (**p < .01).

**TABLE 3:** Levels 8‐PN conjugates and aglycone in the livers

Mouse #	8‐PN total^a^	8‐PN aglycone^b^	Percentage of aglycone
	nmol/g wet tissue		
1	15.47	3.23	20.9
2	17.05	1.62	9.5
3	23.68	1.50	6.3
4	8.45	0.66	7.8
5	20.82	1.18	5.7
6	14.26	2.24	15.7
7	3.90	0.62	15.9
Ave.	14.80	1.58	11.7
SE	2.58	0.32	2.0

Note

Values are derived from 8‐PN detected in the liver with and without deconjugation. The percentages of aglycone to the totals were calculated based on the values obtained for each mouse.

^a^ Total of conjugates and aglycone: with deconjugation.

^b^ Without deconjugation.

### 3.2 Pharmacokinetics of 8‐PN and naringenin in the blood

**FIGURE 2:**
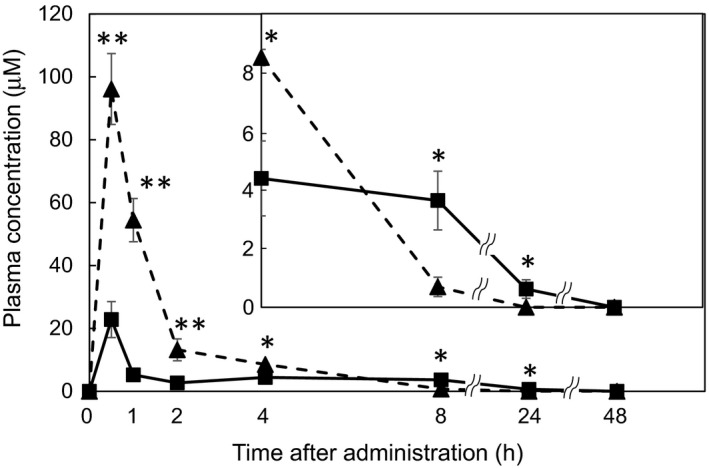
Plasma concentrations of 8‐PN and naringenin after oral administration to mice. Each flavonoid was orally administered at 50 mg/kg BW in a single dose via oral gavage. Plasma samples were collected at 0.5, 1, 2, 4, 8, 24, and 48 h after administration. Inset represents a duplicated enlarged graph of the results from 4 to 48 h. The plasma concentrations of each flavonoid were determined using HPLC–UV after deconjugation treatment. Closed triangle: naringenin, closed square: 8‐PN. Data are presented as the mean ± SE (*n* = 5). Asterisks indicate significant differences between naringenin and 8‐PN at the same time points as determined by using the Mann–Whitney *U* test (*p < .05 and **p < .01, respectively)

The *C*_max_ of 8‐PN (22.8 µM) was lower than that of naringenin (Figure 2). The plasma concentrations of 8‐PN were lower than those of naringenin from 0.5 to 4 h after ingestion. At 8 and 24 h post ingestion, the plasma concentrations of 8‐PN became higher than those of naringenin (Figure 2 inset). The plasma concentrations of naringenin decreased over time, beginning at 0.5 h after ingestion, whereas those of 8‐PN increased 4 h after ingestion and were maintained up to 8 h after ingestion. Only 8‐PN was detected in the plasma 24 h after ingestion. Neither 8‐PN nor naringenin was detected in the plasma 48 h after ingestion.

### 3.3 Binding profiles of 8‐PN to HSA

The appropriateness of the method involving site‐selective HSA‐binding fluorescent probes was confirmed using ibuprofen and phenylbutazone as model drugs. In this study, the inhibition ratio of 100 µM phenylbutazone (which preferentially binds to site I) was demonstrated to be 71.6 ± 0.9% and 23.9 ± 3.5% for sites I and II, respectively. Meanwhile, the inhibition ratio of ibuprofen (which preferentially binds to site II) was demonstrated to be 46.6 ± 2.7% and 81.3 ± 1.0% for sites I and II, respectively. These results were consistent with those of a previous report, indicating that the procedure was appropriate for this study (Er et al., 2013).

**FIGURE 3:**
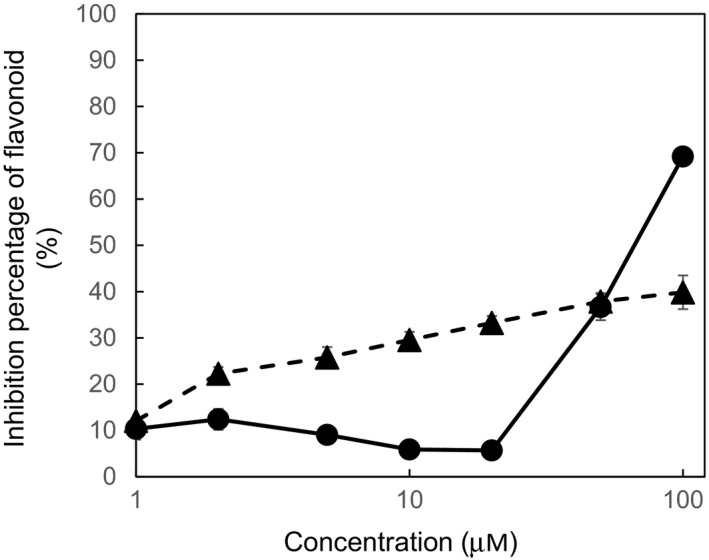
Competition profiles of 8‐PN and 8‐PN‐7G for site I in HSA using the site‐selective HSA‐binding fluorescent probe DNSA. Data are calculated as the competitive inhibition rate (%) of DNSA with flavonoids (*n* = 3, mean ± SE)

The flavonoids tested in this study preferentially bound site I rather than site II (Table 4). The binding affinities of 8‐PN to sites I and II in HSA were stronger than those of naringenin. Furthermore, 8‐PN‐7G had a lower binding affinity for site I than naringenin, whereas aglycone had a higher affinity. However, the binding of 8‐PN to both sites was absent following glucuronidation at the 4′‐position. Additionally, 8‐PN and 8‐PN‐7G at concentrations of 50 µM exhibited comparable inhibition ratios (36.7% and 37.9%, respectively; Figure 3). However, at 20 and 2 µM, as compared to 8‐PN, more 8‐PN‐7G was bound to site I of HSA. However, this difference decreased at concentrations of 1 µM (8‐PN: 10.3%; 8‐PN‐7G: 12.8%). The binding constant values of 8‐PN and 8‐PN‐7G were 2.38 × 10^4^ M and 2.42 × 10^4^ M, respectively.

**TABLE 4:** Competition profiles of 8‐PN and related compounds with site‐selective HSA‐binding fluorescent probes

Aglycone	8‐PN	Naringenin
Site I		
	61.1 ± 0.6*	25.3 ± 0.4
7‐glucuronide	(8‐PN‐7G)	(N7G)
	37.2 ± 0.6*	10.0 ± 3.0
4′‐glucuronide	(8‐PN‐4′G)	(N4′G)
	n.d.^a^	5.2 ± 2.8
Site II		
	28.3 ± 2.8*	8.3 ± 3.9
7‐glucuronide	(8‐PN‐7G)	(N7G)
	n.d.^a^	9.6 ± 4.8
4′‐glucuronide	(8‐PN‐4′G)	(N4′G)
	n.d.^a^	2.4 ± 2.4

Note

Data were calculated as the competitive inhibition rate (%) of the probe binding to flavonoids (*n* = 3, mean ± SE). Asterisks indicate significant differences between 8‐PN and correspondent naringenin as determined by using the Mann–Whitney *U* test (*p < .05).

^a^ The result without competitive inhibition (≤0) with the fluorescent probe is shown as not determined (n.d.).

### 3.4 HEK293 cellular uptake of 8‐PN

**FIGURE 4:**
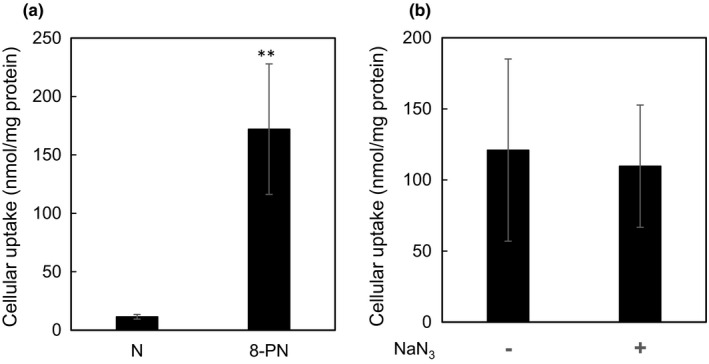
Uptake of 8‐PN and naringenin by HEK293 cells. Cells seeded on 60‐mm dishes were used. Cells were treated with (a) naringenin (N) or 8‐PN (10 µM) for 1 h. Data are presented as the mean ± SE (*n* = 6). (b) Cells were treated with NaN_3_ (100 µM) for 15 min. Then, 8‐PN (10 µM) was added to cells for 1 h. Flavonoid content was quantified using HPLC–UV analysis. Data are presented as the mean ± SE (*n* = 3). Asterisks indicate significant differences as determined by using the Mann–Whitney *U* test (**p < .01)

We also measured the uptake of 8‐PN in HEK293 cells, a model kidney cell line, in which 8‐PN preferentially accumulates (Table 2). We found that, as compared to naringenin, 8‐PN was incorporated into cells and cellular membranes at higher levels (Figure 4a). We then investigated whether 8‐PN was excreted by the ATP‐binding cassette (ABC) transporter, which is reportedly responsible for naringenin elimination (Surya Sandeep et al., 2014). Sodium azide, an inhibitor of this ATPase, did not affect the amount of intracellular 8‐PN (Figure 4b), suggesting that 8‐PN is less susceptible to elimination via this energy‐dependent pathway.

## 4 DISCUSSION

The compound 8‐PN found in hops and beer is known to be a health‐promoting compound owing to its phytoestrogenic activity and other biological functions. However, few studies have demonstrated the pharmacokinetics of 8‐PN in humans and rodents. We elucidated the parameters related to bioavailability of 8‐PN and compared them with those of naringenin.

A previous report demonstrated that prenylation of quercetin at the 8‐position (8‐prenyl quercetin) increased its accumulation in the liver and kidney tissues in mice (Mukai et al., 2013), a phenomenon that is shared by 8‐PN. Tissue accumulation of 8‐PN in the muscles, lungs, pancreas, heart, spleen, and brain tissue was also significantly higher than that of naringenin in mice. Additionally, the amount of 8‐PN in kidneys, muscles, heart, the brain tissues was higher than that of 8‐prenylquercetin under similar feeding conditions (Mukai et al., 2013). Thus, it has been suggested that prenylation of naringenin at the 8′‐position may promote the tissue accumulation of naringenin. This feature may be more pronounced in flavanone type flavonoids (e.g., naringenin) than in flavonol type flavonoids (e.g., quercetin).

Among the tissues and organs investigated in this study, 8‐PN was found to be the most abundant in the kidneys, demonstrating unique characteristics to naringenin, which was found to be abundant in the liver tissues. While several reports have demonstrated that flavonoids are highly accumulated in the liver, the methylated flavonoid tangeretin has been observed to accumulate in the kidney in rat (Hung et al., 2018). It is generally assumed that flavonoids distribute to the tissues in accordance with its drug absorption profile and metabolic pathway. These drugs are abundantly taken up in tissues with developed capillaries via absorptive epithelium. Based on this knowledge, parenchymal cells of the liver (approximately 70% of the liver) or the renal tubular epithelium would be responsible for transporting flavonoids, though there has been no report on the types of cells that contribute to the uptake of flavonoids. Because the liver and kidneys are responsible for drug metabolism, it is presumed that 8‐PN and naringenin would be present in these organs for detoxification and elimination. Other than in the liver and kidneys, passive diffusion (Brand et al., 2006; Murota et al., 2002) is another candidate for flavonoid transport into these tissues. In this case, there may be little differences in the types of cells in each tissue.

To estimate the absorption and elimination rates of 8‐PN in humans, we applied it to a kidney cell line derived from humans (HEK293 cells). The data indicated that 8‐PN in the HEK293 cells had high cellular uptake and low elimination via the ABC transporter. These phenomena may lead to gradual increases in the amount of 8‐PN in the kidneys. Transporters are one of the key factors responsible for the uptake and excretion of flavonoids in the target cell. Although a consensus has not yet been reached regarding the transporters that uptake flavonoids into the cells, it has been reported that hydrophobicity (Murota et al., 2000) and the association with liposome membrane (a model of cellular membrane, Murota et al., 2002) correlate with the cellular uptake of flavonoids. This suggests that passive diffusion is a candidate for flavonoid transport into the cells, and the differences in species between humans and mice are considered to be smaller for the uptake of flavonoid via diffusion than via transporters. The prenyl group can enhance cellular uptake via cellular membranes because of the increase in the hydrophobicity of the mother compound (naringenin). In terms of elimination, ABC transporters (e.g., breast cancer resistance protein or multidrug resistance‐associated protein) contribute to the transportation of flavonoids from the cells (Brand et al., 2006). 8‐PN acts as an ABC transporter inhibitor that reduces ATPase activity (Tan et al., 2014), which may explain its low degree of elimination from HEK293 cells. Although ABC transporters are commonly expressed in the kidneys in both humans and mice, differences in the expression levels in mice and humans would affect the amount of each flavonoid in the cells. However, it is difficult to discuss differences between mice and humans on the elimination of 8‐PN because ABC transporters did not affect 8‐PN transport (Figure 4b). To clarify the elimination of 8‐PN in humans, we have to analyze urine samples, screen for transporters that regulate the cellular accumulation, and discover proteins associated with 8‐PN in the human body.

Another feature of 8‐PN is its relatively high accumulation in skeletal muscles. Our previous report has demonstrated that 8‐PN promotes muscle synthesis and suppresses muscle atrophy (Mukai et al., 2012, 2016). Taken together, the muscular accumulation of 8‐PN may aid the maintenance of skeletal muscle mass.

Although 8‐PN and naringenin were present at lower levels in the brain tissue than in other tissues, 8‐PN was present at higher levels than naringenin. As the permeability of flavonoids into the brain is regulated by the blood–brain barrier (BBB) (Youdim et al., 2004), prenylation of naringenin may have improved its penetration into the BBB. It has also been reported that 8‐PN undergoes phase I and II metabolism of its prenyl group or naringenin skeleton in cells derived from humans (Nikolic et al., 2004, 2006). The observed aglycone ratios in the liver tissues (Table 3) indicate that the conjugated metabolites of 8‐PN are the dominant structures present in mice. To clarify the distribution of the organ‐specific metabolites, it is necessary to determine the composition ratios of phase I and II enzyme metabolites in future studies.

The *C*_max_ of 8‐PN and naringenin in the plasma after single doses was not consistent with the observed tissue distribution (Figure 2). However, 8 h after administration of single doses, the plasma concentrations of 8‐PN were higher than that of naringenin (Figure 2 inset). In addition, a previous report suggested that the long‐term feeding of 8‐PN resulted in higher plasma concentrations than naringenin (Mukai et al., 2012). Furthermore, it has been suggested that prenylflavonoids (e.g., 8‐prenylquercetin and XN) accumulate in the gastrointestinal mucosa (Mukai et al., 2013; Pang et al., 2007), which may result from the gradual transport of 8‐PN (from the intestinal mucosa to the basolateral side) to maintain its circulation. However, further investigation is needed to evaluate the accumulation of 8‐PN in the gastrointestinal tract. As shown in Figure 2, more 8‐PN than naringenin enters the intestinal–hepatic circulation and is reabsorbed, indicating that a longer feeding period may result in increased 8‐PN levels in the body. Co‐administration of prenylflavonoids with proteins (O'Connor et al., 2018) or suspension in propylene glycol liposomes (Yang et al., 2012) has been reported to improve their intestinal absorption. Therefore, technological development to improve the absorption of 8‐PN is needed to apply it in clinical nutrition.

Among the serum proteins, serum albumin plays important role in protein binding for phytochemicals, which is of key importance to tissue distribution of flavonoids in the body. It has been demonstrated that HSA acts as a carrier of flavonoids for blood distribution (Tu et al., 2015) and improves their structural stability (Zinellu et al., 2015). Though we can obtain HSA and mouse serum albumin, we utilized HSA to apply the result to health promotion effects in humans. The affinities of 8‐PN for sites I and II in albumin were stronger than those of naringenin. Although glucuronides weaken their affinity at 100 µM, 8‐PN‐7G retained its affinity for HSA at 37.2%. Furthermore, 8‐PN‐7G retained its affinity up to 1 µM (Figure 3). Meanwhile, N7G did not bind HSA. 8‐PN‐7G has been reported as the predominant metabolite of 8‐PN in human hepatocytes (Nikolic et al., 2006). Several studies have demonstrated that not only naringenin but also other flavonoids interact with HSA in clinical studies and vitro assays (Cao et al., 2019; Murota et al., 2007; Quah et al., 2020). In the clinical study, the binding of quercetin (a major flavonoid found in food), when the volunteers consume a quercetin‐containing food item, onion, has been demonstrated (Murota et al., 2007). The binding constant value of quercetin to HSA was comparable to that of naringenin (Bolli et al., 2010). The binding constant values of 8‐PN and 8‐PN‐7G obtained here were 2.38 × 10^4^ and 2.42 × 10^4^, respectively (Figure 4). Although the determination method is different, these values are similar to those of naringenin (Tu et al., 2015). These data indicate that 8‐PN binds to HSA in vivo.

The folding or conformation of proteins regulates their biological functions (Ishima et al., 2008). The effect of chemicals (Chamani et al., 2003; Chamani & Heshmati, 2008; Chamani & Moosavi‐Movahedi, 2006) or biological components such as proteins (Sadeghzadeh et al., 2020) on protein conformation has been reported. It has been shown that the binding of flavonoids to HSA modifies its conformation and stability (Barreca et al., 2017). Binding of certain ligands such as amino acids (Beigoli et al., 2019) and fatty acids (Ishima et al., 2008) to HSA affects the binding property or biological properties of drugs. This information suggests that 8‐PN and 8‐PN‐7G may impact the folding, conformation, and biological function of HSA.

Though bioavailability properties indeed differ from species to species, and data from mice cannot be applied directly to humans, it may be assumed that a higher affinity of 8‐PN to HSA contributes to increasing the blood concentration of 8‐PN and, consequently, its tissue distribution. It has been reported that albumin demonstrates 70%–80% primary amino acid sequence homology between species (Kosa et al., 1998; Theodore Peters, 1996), and they show a wide variety of binding properties to ligands in different species (Pistolozzi & Bertucci, 2008; Yanagisawa et al., 1997). It is the limitation of our research that the binding property of each flavonoid to HSA cannot directly reflect their pharmacokinetics in mice. To cover the gap in species, it is necessary to use novel experimental models (e.g., HSA transgenic mouse model) (Sheng et al., 2016).

8‐PN tissue distribution and pharmacokinetics in mice and its association to HSA and cellular uptake in a human‐derived cell line were demonstrated in this study. Our results demonstrated that 8‐PN bioavailability was obviously different from that of naringenin. Since 8‐PN showed a higher accumulation in mouse tissues and higher cellular uptake in HEK293, 8‐PN is suspected to have higher bioavailability than naringenin, irrespective of the model organism. Our data suggest the necessity to investigate its excretion in the future. To use 8‐PN as a health‐promoting nutrient, it is important to obtain data about not only the health promotion effect but also its potential toxicity if its elimination is slow. A limitation of this study is that the obtained data cannot be compared between humans and mice; thus, it is necessary to consider the differences in absorption and metabolism of flavonoids between rodents and humans (Bai et al., 2020; Brand et al., 2010; Ning et al., 2015). Despite these limitations, this study provided useful information for determining target tissues in which 8‐PN exerts a health‐promoting effect.

## CONFLICT OF INTEREST

This study was partly funded by Dicel Corporation. Yuichi Ukawa and Kenichi Oe are employees of Dicel Corporation.

## AUTHOR CONTRIBUTIONS

**Yoshiaki Tanaka:** Data curation (supporting). **Hitomi Okuyama:** Data curation (supporting). **Miyu Nishikawa:** Resources (lead). **Shin‐ichi Ikushiro:** Resources (lead); Supervision (supporting); Writing – review & editing (supporting). **Mayumi Ikeda:** Data curation (supporting); Formal analysis (equal). **Yu Ishima:** Formal analysis (supporting); Methodology (supporting); Writing – review & editing (supporting). **Yuichi Ukawa:** Resources (supporting). **Kenichi Oe:** Resources (supporting). **Junji Terao:** Funding acquisition (supporting); Project administration (supporting). **Rie Mukai:** Conceptualization (lead); Data curation (lead); Formal analysis (lead); Funding acquisition (lead); Investigation (lead); Project administration (lead); Supervision (lead); Validation (lead); Writing – original draft (lead); Writing – review & editing (lead).

## Data Availability

The data that support the findings of this study are available from the corresponding author upon reasonable request.
